# Fragment Linking and Optimization of Inhibitors of the Aspartic Protease Endothiapepsin: Fragment‐Based Drug Design Facilitated by Dynamic Combinatorial Chemistry

**DOI:** 10.1002/anie.201603074

**Published:** 2016-07-12

**Authors:** Milon Mondal, Nedyalka Radeva, Hugo Fanlo‐Virgós, Sijbren Otto, Gerhard Klebe, Anna K. H. Hirsch

**Affiliations:** ^1^ Stratingh Institute for ChemistryUniversity of GroningenNijenborgh 79747AGGroningenThe Netherlands; ^2^ Institute of Pharmaceutical ChemistryMarbach Weg 635032MarburgGermany; ^3^ Centre for Systems Chemistry, Stratingh Institute for ChemistryUniversity of GroningenNijenborgh 49747AGGroningenThe Netherlands

**Keywords:** dynamic combinatorial chemistry, fragment**-**based drug design, inhibitors, proteases, X**-**ray diffraction

## Abstract

Fragment‐based drug design (FBDD) affords active compounds for biological targets. While there are numerous reports on FBDD by fragment growing/optimization, fragment linking has rarely been reported. Dynamic combinatorial chemistry (DCC) has become a powerful hit‐identification strategy for biological targets. We report the synergistic combination of fragment linking and DCC to identify inhibitors of the aspartic protease endothiapepsin. Based on X‐ray crystal structures of endothiapepsin in complex with fragments, we designed a library of bis‐acylhydrazones and used DCC to identify potent inhibitors. The most potent inhibitor exhibits an IC_50_ value of 54 nm, which represents a 240‐fold improvement in potency compared to the parent hits. Subsequent X‐ray crystallography validated the predicted binding mode, thus demonstrating the efficiency of the combination of fragment linking and DCC as a hit‐identification strategy. This approach could be applied to a range of biological targets, and holds the potential to facilitate hit‐to‐lead optimization.

Over the past decade, fragment‐based drug design (FBDD) has emerged as a novel paradigm in drug discovery and it has been applied to a growing number of biological targets.^1^, ^2^, ^3^ FBDD has higher hit rates than high‐throughput screening and enables coverage of the chemical space using smaller libraries.^2^ Since its inception in the mid‐1990s,^4^ FBDD has expanded tremendously and various pharmaceutical companies have used FBDD to develop more than 18 drug candidates that are now in clinical trials.^5^

After the identification of fragment hits by various screening techniques, the hits are optimized to lead compounds and drug candidates by fragment growing, linking, and/or merging. Fragment growing, on the one hand, has become the favorite optimization strategy,^6^, ^7^ even though it involves cycles of iterative design, synthesis and validation of the binding mode of each derivative. To overcome this drawback, we have previously reported the combination of fragment growing and dynamic combinatorial chemistry (DCC) to accelerate drug discovery.^8^ Fragment linking, on the other hand, is attractive because of the potential for super‐additivity (an improvement in ligand efficiency (LE) rather than mere maintenance of LE). The first example of fragment linking was reported by Fesik and co‐workers.^4^, ^9^ Since then, a few studies demonstrating the efficiency of fragment linking of low‐affinity fragments to produce higher‐affinity ligands have been reported.^10^, ^11^ The challenge lies in preserving the binding modes of the fragments in adjacent pockets whilst identifying a linker featuring an optimal fit.^12^, ^13^

In addition to FBDD, DCC^14^, ^15^, ^16^, ^17^, ^18^ and dynamic ligation screening (DLS)^19^, ^20^, ^21^, ^22^ are powerful strategies for identifying/optimizing hit compounds for biological targets. In a dynamic combinatorial library (DCL), the bonds between the building blocks are reversible and are continuously being made and broken. Addition of the target protein leads to re‐equilibration as one or more library components are bound to the protein, resulting in amplification of the strongest binder(s) from the DCL. In DLS, formation of a reversible covalent bond between a directing probe and a nucleophilic fragment enables the detection of low‐affinity ligands while measuring at micromolar concentrations.

We therefore envisaged the potentially synergistic combination of fragment linking and DCC as an efficient hit‐identification/optimization strategy. In this work, we combined fragment linking and bis‐acylhydrazone‐based DCC to identify inhibitors for endothiapepsin, which belongs to the notoriously challenging family of pepsin‐like aspartic proteases.^23^

Aspartic proteases are found in fungi, vertebrates, plants, and retroviruses such as HIV. This class of enzymes play a causative role in important diseases such as malaria, Alzheimer's disease, hypertension, and AIDS.^23^ Owing to its high similarity with these drug targets, endothiapepsin has been used as a model enzyme for mechanistic studies^24^, ^25^, ^26^ and for the discovery of inhibitors of renin^27^ and β‐secretase.^28^ Endothiapepsin is a robust enzyme, which remains active for more than 20 days at room temperature, is readily available in large quantities, and crystallizes easily, thus making it a useful representative for aspartic proteases.^18^ Pepsin‐like aspartic proteases are active as monomers and consist of two structurally similar domains, each of which donates an aspartic acid residue to the catalytic dyad (D35 and D219 in endothiapepsin), which hydrolyzes the peptide bond of the substrate through nucleophilic attack by a catalytic water molecule.

Although bis‐acylhydrazone‐based DCC has been reported,^29^ there are no reports of fragment linking using DCC. Herein, we describe the combination of fragment linking/optimization and DCC to efficiently afford ligands for inhibiting the aspartic protease endothiapepsin.

We chose X‐ray crystal structures of endothiapepsin in complex with acylhydrazones **1** and **2** (PDB IDs: 4KUP and 3T7P, respectively) as a starting point for fragment linking (Figure 1). We had previously identified **1** and **2** as hits from an acylhydrazone‐based DCL using the synergistic combination of de novo SBDD and DCC.^18^ Our hits **1** and **2** displayed IC_50_ values of 12.8 μm and 14.5 μm and ligand efficiencies (LEs) of 0.27 and 0.29, respectively, against endothiapepsin. Both hits displayed alternative binding modes with the catalytic dyad: either through a water‐mediated interaction or through direct interaction, with displacement of the lytic water molecule. Fragments **1** and **2** occupy the S1 and S2 or S1 and S2′ pockets, respectively (Figure 2 a).

**Figure 1:**
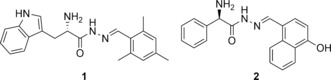
Structures of hits **1** and **2**.

**Figure 2:**
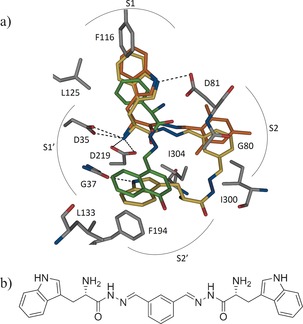
a) Superimposition of the crystallographically determined binding modes of **1** (C: orange) and **2** (C: green) (PDB IDs: 4KUP and 3T7P, respectively) with a putative bis‐acylhydrazone inhibitor (C: yellow). b) Chemical structure of the modeled bis‐acylhydrazone shown in Figure 2 a.^33^

We envisaged the linking of **1** and **2** to afford an inhibitor that should occupy the S1, S1′, S2, and S2′ pockets of endothiapepsin and benefit from numerous protein–ligand interactions, while maintaining/improving the LE. With the help of the molecular‐modeling software Moloc,^30^ we linked the mesityl moiety of **1** to the naphthyl moiety of **2** through an acylhydrazone linker, which resides at the junction of the S2 and S2′ pockets and appeared to be a suitable linker. Acylhydrazone‐based DCC is attractive for medicinal chemistry‐based projects because the resulting products feature both H‐bond donors and H‐bond acceptors and are stable enough as drug candidates under acidic and physiological conditions. In our previous studies, we demonstrated that acylhydrazone‐based DCC is compatible with endothiapepsin.^8^, ^18^ Inspection of known co‐crystal structures of endothiapepsin^31^ and hotspot analysis^32^ of the active site suggested that both aromatic and aliphatic moieties can be hosted in the S2′ pocket, since they benefit from hydrophobic contacts with residues G37, L133, and F194. Based on our molecular‐modeling studies and evaluation of synthetic accessibility, we designed and optimized a series of bis‐acylhydrazone‐based inhibitors of endothiapepsin. A superimposition of a modeled potential bis‐acylhydrazone‐based inhibitor and the parent fragments is shown in Figure 2. All of the bis‐acylhydrazones form H‐bonding interactions with the catalytic dyad and most of them occupy the S1, S2, S1′, and S2′ pockets, and maintain the binding mode of fragments **1** and **2**.

Retrosynthetic analysis of the designed bis**‐**acylhydrazones led to isophthalaldehyde (**3**) and nine hydrazide building blocks **(4**–**12**) for DCC (Scheme 1). Whereas the bis**‐**aldehyde **3** and hydrazides **10**–**12** are commercially available, we synthesized hydrazides **4**–**9** from their corresponding methyl esters through treatment with hydrazine monohydrate (60**–**90 **%** yield, see Scheme S1 in the Supporting Information).

**Scheme 1:**
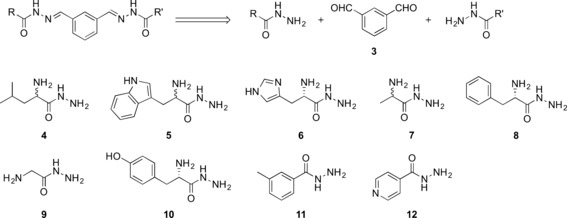
Structures and retrosynthetic analysis of the designed bis‐acylhydrazone inhibitors and their corresponding aldehyde (**3**) and hydrazide (**4**–**12**) precursors.

We set up a DCL with bis‐aldehyde **3** and the nine hydrazides **4**–**12**, which has the potential to produce 78 bis‐acylhydrazones (excluding *E*/*Z* isomers) and 12 mono‐acylhydrazones. To facilitate the analysis, we divided the library into two sub‐libraries. We used reversed‐phase HPLC and LC–MS to analyze and identify the best binders from the DCLs and we employed aniline as a nucleophilic catalyst to ensure that the equilibrium is established faster than in the absence of a catalyst.

The first library, DCL‐1, consisted of the four hydrazides **5**, **6**, **10**, and **12** (100 μm each), and bis‐aldehyde **3** (50 μm) in presence of 10 mm aniline and 2 % DMSO in 0.1 m sodium acetate buffer at pH 4.6, thus resulting in the formation of 15 potential homo‐ and hetero‐bis‐acylhydrazones (excluding *E*/*Z* isomers) and five mono‐acylhydrazones in equilibrium with the initial building blocks. We were able to detect all of the homo‐ and hetero‐bis‐acylhydrazones by LC–MS analysis. Upon the addition of endothiapepsin, we observed amplification of the bis‐acylhydrazones **13** and **14** by more than three times compared to the blank reaction (Figure 3 and Figure S1 in the Supporting Information). We set up the second library, DCL‐2, using the five hydrazides **4**, **7**, **8**, **9**, and **11** (100 μm each), and bis‐aldehyde **3** (50 μm) under the same conditions, giving rise to the formation of 28 potential homo‐ and hetero‐bis‐acylhydrazones (excluding *E*/*Z* isomers) and seven mono‐acylhydrazones in equilibrium with the initial building blocks. Upon addition of the protein, bis‐acylhydrazones **15** and **16** were amplified by a factor of more than two compared to the blank reaction (Figure 3 and Figure S2 in the Supporting Information). We also constructed a large library, DCL‐3, using all nine hydrazides (**4**–**12**) and bis‐aldehyde **3** and observed amplification of the previously observed bis‐acylhydrazones **13**, **14**, and **16** along with bis‐acylhydrazones **17** and **18** (Figure 3 and S3 in the Supporting Information). We identified a total of two homo‐ (**13** and **16**) and four hetero‐ (**14**, **15**, **17** and **18**) bis‐acylhydrazones from the three libraries DCL‐1–3 (Figure 3).

**Figure 3:**
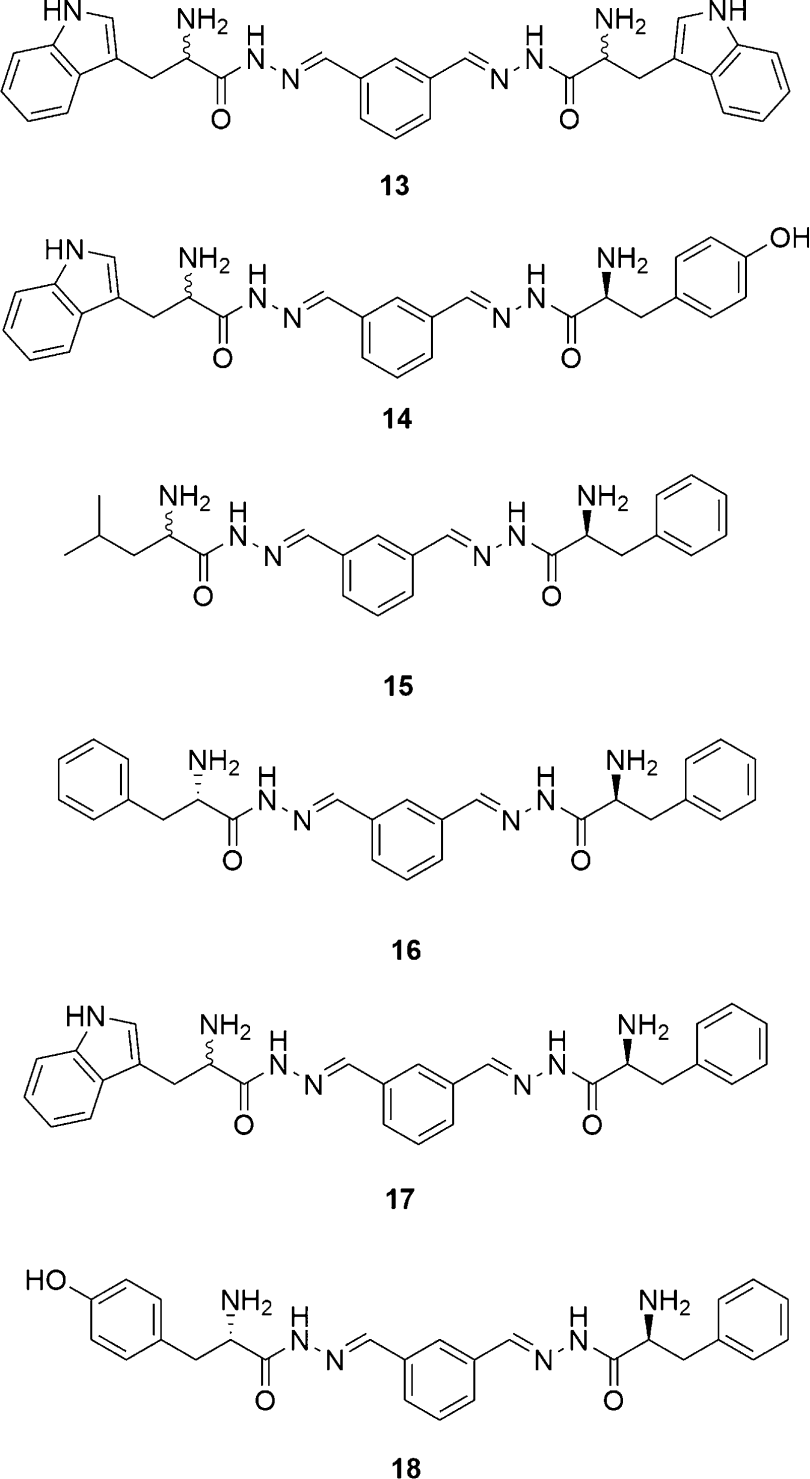
Chemical structures of the bis‐acylhydrazones identified from three DCLs using LC–MS analysis.

To determine the biochemical activity of the amplified bis‐acylhydrazones, we synthesized the two homo‐bis‐acylhydrazones **13** and **16** from their corresponding hydrazides **5** and **8** and the bis‐aldehyde **3** (see Schemes S2 and S3 in the Supporting Information). We determined their inhibitory potency by applying a fluorescence‐based assay adapted from an assay for HIV protease.^34^ Biochemical evaluation confirmed the results of our DCC experiments, which were analyzed by LC–MS. Bis‐acylhydrazones **13** and **16** indeed inhibit the enzyme with IC_50_ values of 0.054 μm and 2.1 μm, respectively (see Figure 4, and Figures S4 and S5 in the Supporting Information). The potency of the best inhibitor was increased 240‐fold compared to the parent hits. The experimental Gibbs free energies of binding (Δ*G*) and LEs, derived from the experimental IC_50_ values using the Cheng–Prusoff equation,^35^ are Δ*G*(**13**)=−49 kJ mol^−1^, Δ*G*(**16**)=−34 kJ mol^−1^, and LE(**13**)=0.29, LE(**16**)=0.25, which represents an improvement in Δ*G* values while preserving the LEs compared to the parent fragments (Table 1).

**Figure 4:**
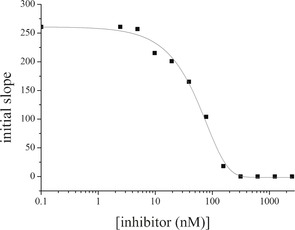
IC_50_ inhibition curve of **13** (IC_50_=54.5±0.5 nm) measured in duplicate; the errors are given as the standard deviation (SD).

**Table 1:** The IC_50_ values, ligand efficiencies (LE), and calculated and experimental Gibbs free energies of binding (Δ*G*) for the parent fragments and bis‐acylhydrazone inhibitors.

Inhibitors	IC_50_ [μm]	*K*_i_ [μm]	Δ*G* ^[a]^ [kJ mol^−1^]	LE^[a]^
1	12.8±0.4	6±0.2	−30	0.27
2	14.5±0.5	7±0.2	−30	0.29
13	0.054±0.0005	0.0254±0.0002	−49	0.29
16	2.1±0.1	0.98±0.05	−34	0.25

[a] The Gibbs free energies of binding (Δ*G*) and the ligand efficiencies (LEs) were derived from the experimentally determined IC_50_ values.

To validate the predicted binding mode of the linked fragments, we soaked crystals of endothiapepsin with the most potent inhibitor (**13**) and determined its crystal structure (PDB ID: 5HCT) in complex with endothiapepsin at 1.36 Å resolution. **13** binds to the S1, S1′, and S2 pockets and addresses the catalytic dyad through its α‐C amino group (Figure 5 a). A part of this bis‐acylhydrazone is not visible in the electron‐density map, thus implying disorder of this substituent across multiple conformational states, which is in line with our modeling studies. In two plausible poses, the unresolved portion of bis‐acylhydrazone **13** would be oriented towards the S2′ and S6 pockets of the enzyme or remain solvent‐exposed (Figure 5 b).

**Figure 5:**
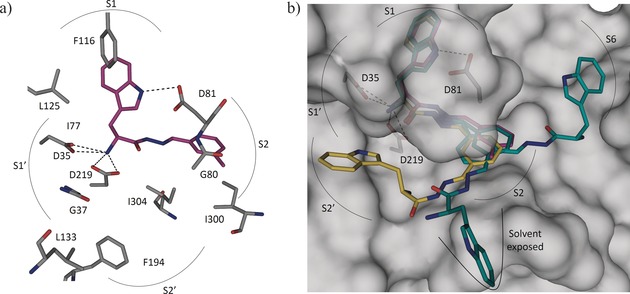
a) X‐ray crystal structure of endothiapepsin co‐crystallized with bis‐acylhydrazone **13** (PDB ID: 5HCT). b) Superimposition of the crystal structure (violet) and modeled structures (yellow and cyan) of **13**.^33^

The detailed binding mode of bis‐acylhydrazone **13** is shown in Figure 5 a. The visible portion of **13** preserves the binding mode of the initial hit **1** and forms four charged H bonds with the catalytic dyad through its α‐C amino group, as well as an H bond with the carboxylate group of D81 through the NH of the indolyl moiety, which is accommodated in the S1 pocket and engaged in offset π–π stacking and CH–π interactions with F116 and L125, respectively. The phenyl group of **13** binds in the S2 pocket and is involved in hydrophobic interactions with I300 and I304. Like the mesityl group of **1**, the phenyl group of **13** also engages in an amide–π interaction with the peptide bond connecting residues G80 and D81. The phenyl moiety in **13** is connected with two imine functionalities, thus making the aromatic ring electron‐deficient, which presumably strengthens the amide–π interaction compared to the electron‐rich mesityl group in **1**.^36^

In this study, we have demonstrated for the first time that the synergistic combination of fragment linking and DCC is a powerful and efficient strategy for accelerating hit identification and optimization to afford inhibitors of the aspartic protease endothiapepsin. We exploited LC–MS analysis to identify the best binders directly from the DCLs. The best inhibitor exhibits an IC_50_ value of 54 nm, representing a 240‐fold improvement in potency. Subsequent soaking experiments validated our in silico fragment linking. Our strategic combination of computational and analytical methods holds great promise for accelerating drug development for this challenging class of proteases, and it could afford useful new lead compounds. This approach could be also extended to a large number of other protein targets.

## Supporting information

As a service to our authors and readers, this journal provides supporting information supplied by the authors. Such materials are peer reviewed and may be re‐organized for online delivery, but are not copy‐edited or typeset. Technical support issues arising from supporting information (other than missing files) should be addressed to the authors.

Supplementary
