# Supplementary material for: Fragment Linking and Optimization of Inhibitors of the Aspartic Protease Endothiapepsin: Fragment‐Based Drug Design Facilitated by Dynamic Combinatorial Chemistry
Source: Angew Chem Int Ed Engl. 2016 Jul 12;55(32):9422–6. doi: 10.1002/anie.201603074 (PMC5113778; doi:10.1002/anie.201603074)
Supplement: Supplementary file 1 — Supplementary [file ANIE-55-9422-s001.pdf]

Angewandte Chemie
Eine Zeitschrift der Gesellschaft Deutscher Chemiker

Supporting Information

Fragment Linking and Optimization of Inhibitors of the Aspartic Protease Endothiapepsin: Fragment-Based Drug Design Facilitated by Dynamic Combinatorial Chemistry

*Milon Mondal, Nedyalka Radeva, Hugo Fanlo-Virgós, Sijbren Otto, Gerhard Klebe, and Anna K. H. Hirsch**

anie_201603074_sm_miscellaneous_information.pdf

Table of Contents

Schemes S1–S3	S2
Figures S1–S5	S3
Formation and analysis of bis-acylhydrazone-based DCLs	S6
Inhibition assay	S7
Modeling studies	S8
Synthetic procedures	S9
NMR spectra	S13
UPLC chromatograms	S19
References	S20

Schemes

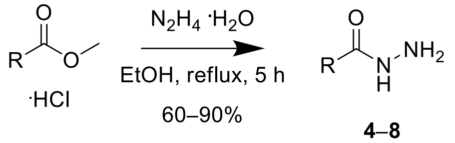

Scheme S1. Synthesis of hydrazides from the corresponding methyl esters.

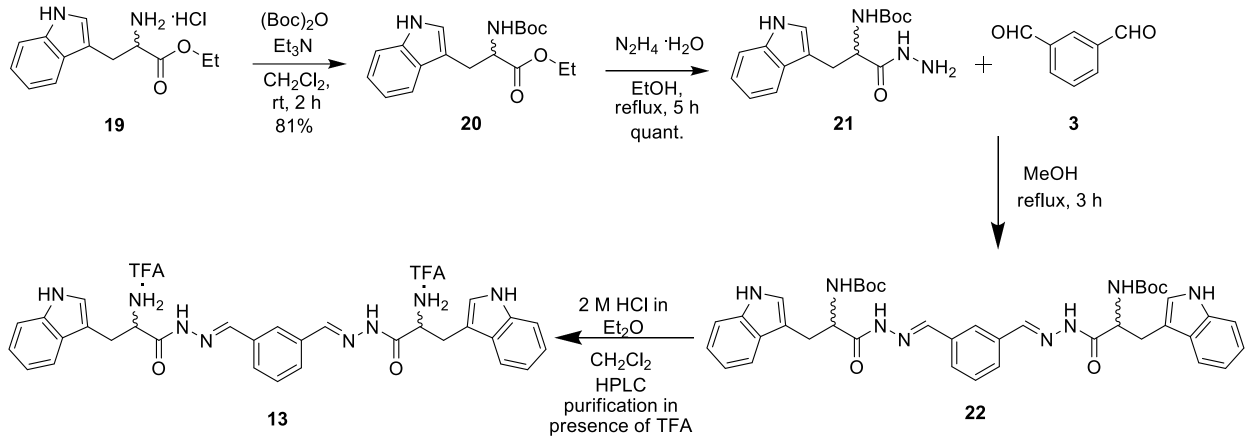

Scheme S2. Synthesis of bis-acylhydrazone **13**.

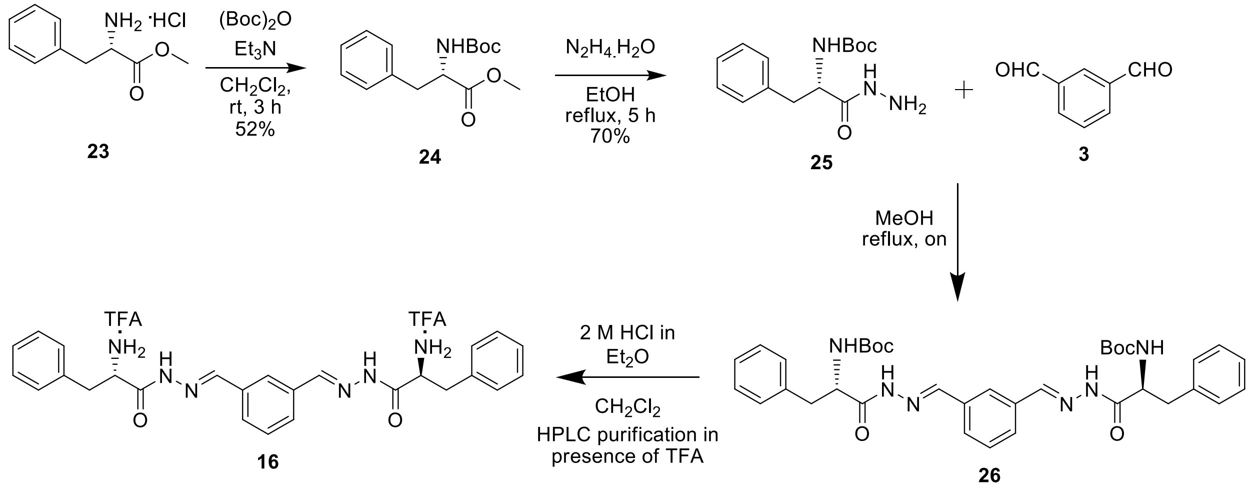

Scheme S3. Synthesis of bis-acylhydrazone **16**.

Figures

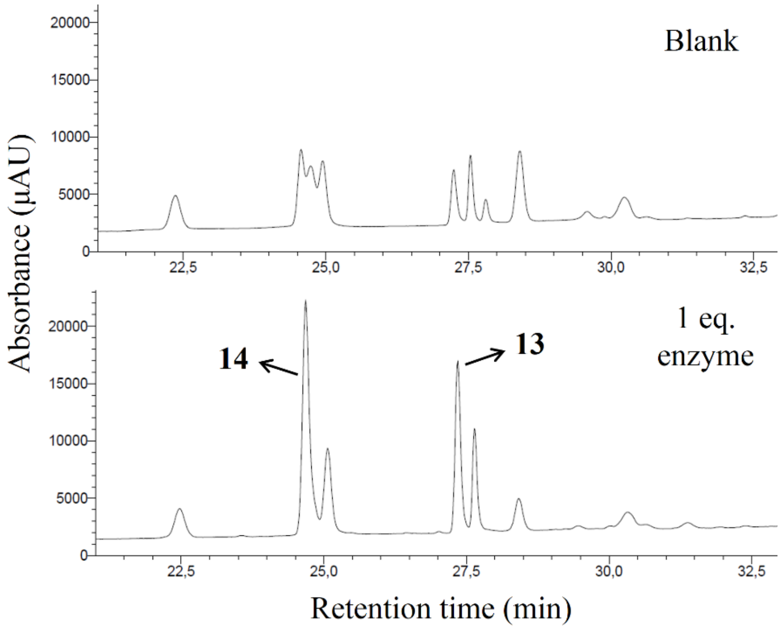

Figure S1. HPLC chromatogram of aniline-catalyzed bis-acylhydrazone formation (DCL-1) using hydrazides **5**, **6**, **10** and **12** (100 µM each) and bis-aldehyde **3** (50 µM) in absence of enzyme (blank, top chromatogram) and in presence of 1 eq. of endothiapepsin (bottom chromatogram). DCL established in the presence of 10 mM aniline. Bis-acylhydrazones **13** and **14** are clearly amplified.

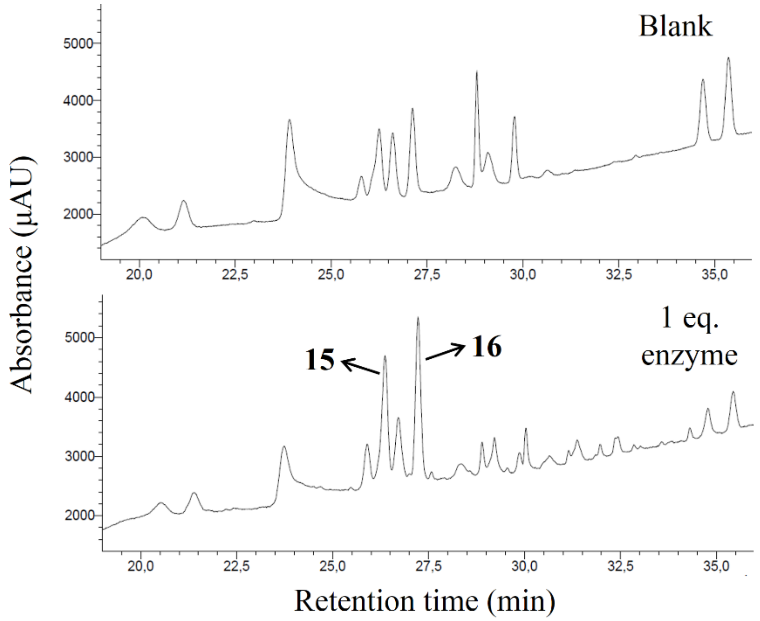

Figure S2. HPLC chromatogram of aniline-catalyzed bis-acylhydrazone formation using hydrazides **4, 7, 8, 9** and **11** (100 µM each) and bis-aldehyde **3** (50 µM) in absence of enzyme (blank, top chromatogram) and in presence of 1 eq. of endothiapepsin (bottom chromatogram). DCL established in the presence of 10 mM aniline. Bis-acylhydrazones **15** and **16** are clearly amplified.

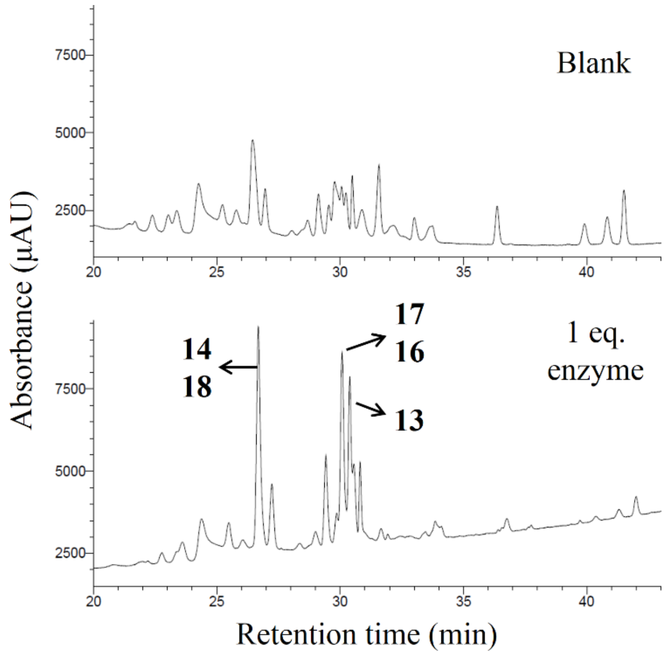

Figure S3. HPLC chromatogram of aniline-catalyzed bis-acylhydrazone formation using hydrazides **4–12** (100 µM each) and bis-aldehyde **3** (50 µM) in absence of enzyme (blank, top chromatogram) and in presence of 1 eq. of endothiapepsin (bottom chromatogram). DCL established in the presence of 10 mM aniline. Bis-acylhydrazones **13**, **14**, **16**, **17** and **18** are clearly amplified.

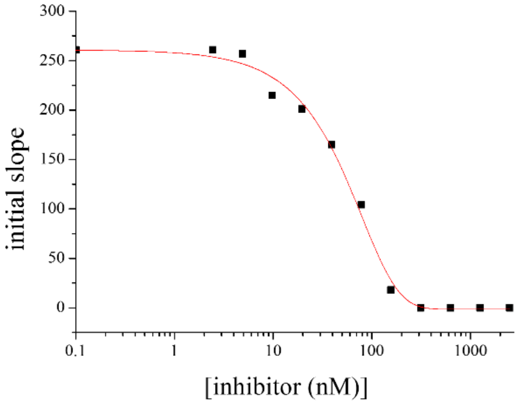

Model DoseResp
Equation y = A1 + (A2-A1)/(1 + 10^((LOGx0-x)*p))

	Value	Standard Error
A1	-1.51869	5.02597
A2	831.57594	817.03148
LOGx0	-47.91053	99.23694
p	-0.00704	0.0019

IC_{50} = 55 nM

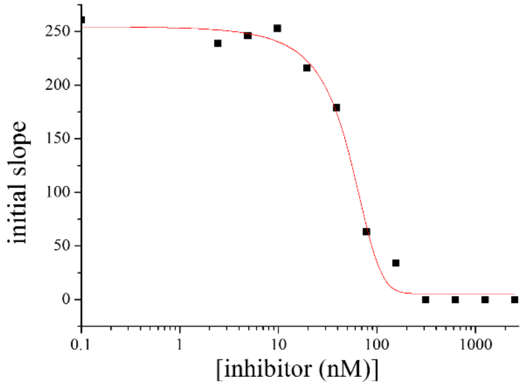

Model DoseResp
Equation y = A1 + (A2-A1)/(1 + 10^((LOGx0-x)*p))

	Value	Standard Error
A1	5.51189	5.68752
A2	287.1057	29.00529
LOGx0	48.56032	7.24237
p	-0.0182	0.00425

IC_{50} = 54 nM

IC_{50} = 54 ± 0.5 nM

Figure S4. IC_{50} inhibition curves of **13** (IC_{50} = 54.5 ± 0.5 nM). It was measured in duplicate and the errors are given in standard deviations (SD), 26 experiments were performed for each measurement and only six experiments were considered to calculate the initial slope (*n* = 6), 11 different concentrations of inhibitor were used starting at 2.5 µM.

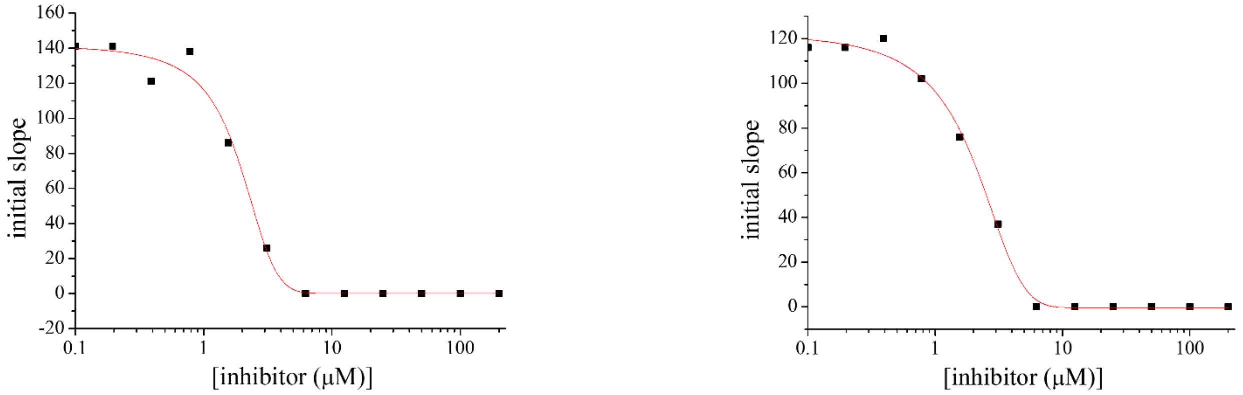

Model	DoseResp	
Equation	y = A1 + (A2-A1)/(1 + 10^((LOGx0-x)*p))	
	Value	Standard Error
A1	0.1025	2.98723
A2	153.25768 13.0935	
LOGx0	1.85643	0.21357
p	-0.58181	0.13153

IC_{50} = 2.0 μM

Model	DoseResp	
Equation	y = A1 + (A2-A1)/(1 + 10^((LOGx0-x)*p))	
	Value	Standard Error
A1	-0.41512	1.43934
A2	153.3503	17.35671
LOGx0	1.64277	0.33551
p	-0.35623	0.05607

IC_{50} = 2.2 μM

IC_{50} = 2.1 ± 0.1 μM

Figure S5. IC_{50} inhibition curves of **16** (IC_{50} = 2.1 ± 0.1 μM). It was measured in duplicate and the errors are given in standard deviations (SD), 26 experiments were performed for each measurement and only six experiments were considered to calculate the initial slope (*n* = 6), 11 different concentrations of inhibitor were used starting at 250 μM.

General procedure for the formation and analysis of the DCLs

Hydrazides (2 μL each, 50 mM in DMSO (**5**, **6**, **10** and **12** for DCL-1; **4**, **7**, **8**, **9** and **11** for DCL-2 and **4–12** for DCL-3)), bis-aldehyde **3** (1 μL, 50 mM in DMSO), aniline (10 μL, 1 M in DMSO) were added to sodium acetate buffer (980 μL, 0.1 M, pH 4.6). The DCL was allowed to stand at room temperature with occasional shaking and the progress of the reaction was monitored by RP-HPLC to establish the blank composition until the relative concentrations of acylhydrazones became constant. After 20 h, the pH of the DCLs (500 μL) was raised to 8 by the addition of NaOH (20 μL, 2 M in water) and acetonitrile (500 μL) was added. LC-MS analysis of the DCL verified the formation of all of the homo-, hetero-bis-acylhydrazones and mono- acylhydrazones.

Endothiapepsin (1 eq., 14 μL, 0.88 mM in 0.1 M sodium acetate buffer, pH 4.6) was added to the DCLs (236 μL). After 20 h, the pH of the DCL was raised to 8 by the addition of NaOH (10 μL, 2 M in water), and acetonitrile (250 μL) was added followed by vortexing and centrifugation

(10,000 g, 3 min) to denature and separate the enzyme from the DCLs. HPLC analysis of the supernatants was performed, and the trace was compared with that of the blank reaction.

HPLC conditions

DCL-1 and DCL-2:

Column, Waters symmetry C8, 3.5 µm, 150 mm × 4.6 mm; flow rate, 0.5 mL min^{-1}; wavelength, 254 nm; temperature, 23 ºC; injection volume, 10 µL; gradient, H_2O/MeCN (0.1% formic acid (FA)) at 95% for 10 min followed by 95% to 5% over 50 min.

DCL-3:

Column, Waters symmetry C8, 3.5 µm, 150 mm × 4.6 mm; flow rate, 0.5 mL min^{-1}; wavelength 254 nm; temperature, 23 ºC; injection volume, 10 µL; gradient, H_2O/MeCN (0.1% FA) at 95% for 10 min followed by 95% to 5% over 70 min).

LC-MS conditions

A Shimadzu LC-system (Shimadzu-SIL-20AC) was coupled to a Sciex API 3000 triple quadrupole mass spectrometer equipped with an ion spray source (Applied Biosystems, MDS-SCIEX, Toronto, Canada) was used. Mass spectra (positive mode) were obtained in profile scan mode from mass 100 till 800 using the following conditions: DP=46V; FP=160V; TEM=450 °C.

Inhibition Assay:

Endothiapepsin was purified from Suparen® (kindly provided by DSM Food Specialties) by exchanging the buffer to sodium acetate buffer (0.1 M, pH 4.6) using a Vivaspin 500 with a molecular weight cutoff at 10,000 Da. The measurement of the absorption at 280 nm, assuming an extinction coefficient of 1.15 for 1 mg/mL solutions, afforded the protein concentration.[3]

Stock solutions (250 µM and 20 mM in DMSO for **13** and **16**, respectively) were prepared. As substrate, Abz-Thr-Ile-Nle-*p*-nitro-Phe-Gln-Arg-NH_2 (purchased from Bachem) was used for the

inhibition studies. The assay was performed with flat bottom 96-well microplates (purchased from Greiner Bio-One) using a Synergy Mx microplate reader at an excitation wavelength of 337 nm and an emission wavelength of 414 nm. The K_m of the substrate toward endothiapepsin is known, 1.6 μM.[4] The assay buffer (0.1 M sodium acetate buffer, pH 4.6, containing 0.001% Tween 20) was premixed with the substrate and the bis-acylhydrazones identified by LC-MS measurements; endothiapepsin was added directly before the measurement. The final reaction volume was 200 μL containing 0.4 nM endothiapepsin, 1.8 μM substrate and 2.5 μM (**13**) and 200 μM (**16**) bis-acylhydrazones. In the same way, blanks were prepared using DMSO instead of the bis-acylhydrazone stock solution. As the substrate is fluorogenic, during the measurement, the fluorescence increases because of substrate hydrolysis by endothiapepsin. The initial slopes of the fluorescence in the bis-acylhydrazone-containing wells were compared to the initial slope of the blanks for data analysis. Each compound was measured in duplicate. The final result represents the average of both measurements.

Modeling studies

Two X-ray crystal structures of complexes of endothiapepsin (PDB codes: 4KUP and 3T7P) were used for our modeling.[1] Several bis-acylhydrazones were designed using fragment linking. The energy of the system was minimized using the MAB force field as implemented in the computer program MOLOC,[5] whilst keeping the protein coordinates fixed for the PDB code: 4KUP. In all cases, the bis-acylhydrazones address the catalytic dyad directly via hydrogen-bonding interactions. Taking inspiration from the co-crystal structures of endothiapepsin with eleven fragments,[4] as well as from hot-spot analysis[6] of the active site of endothiapepsin, several bis-acylhydrazones with different aromatic and aliphatic substituents were designed and subsequent energy minimization (MAB force field) was done using MOLOC. All types of interactions (hydrogen bonds and lipophilic interactions) between designed bis-acylhydrazones and protein were measured in MOLOC.

Synthetic Procedures

General Experimental Details

Starting materials and reagents were purchased from Aldrich or Acros. Yields refer to analytically pure compounds and have not been optimized. All solvents were reagent-grade and if necessary,

SPS-grade. Column chromatography was performed on silica gel (Silicycle® SiliaSepTM 40-63 μM 60 Å). TLC was performed with silica gel 60/Kieselguhr F254. Solvents used for the column chromatography were dichloromethane and methanol. ^{1}H-, ^{13}C- and ^{19}F-NMR spectra were recorded at 400 MHz on a Varian AMX400 spectrometer (400 MHz for ^{1}H, 101 MHz for ^{13}C and 376 MHz for ^{19}F) at 25 °C. Acylhydrazone NMR spectra consist of both *E* and *Z* isomers. Chemical shifts (δ) are reported relative to the residual solvent peak. Splitting patterns are indicated as (s) singlet, (d) doublet, (t) triplet, (q) quarted, (m) multiplet, (br) broad. The coupling constants (J) are given in Hz. High-resolution mass spectra (HR-MS) were recorded with a FTMS orbitrap (Thermo Fisher Scientific) mass spectrometer. FT-IR were measured on a PerkinElmer FT-IR spectrometer. Melting points were measured on a Stuart® SMP11 melting point apparatus. UPLC conditions: column, ACQUITY UPLC® HSS T3 1.8 μm, 150 mm × 2.1 mm; flow rate, 0.3 mL min^{-1}; wavelength, 254 nm; temperature, 35 °C; gradient method, water/acetonitrile (0.1% FA) 95:5 → 30:70 in 17 min followed by 30:70 → 5:95 in 5 min.

Synthesis of the hydrazides:

General procedure for hydrazide formation from the corresponding methyl/ethyl ester (GP1):

To a solution of the methyl/ethyl ester hydrochloride (1 eq.) in ethanol (4 mL), was added hydrazine monohydrate (8 eq.). The reaction mixture was heated to reflux for 5 h. The reaction mixture was concentrated *in vacuo*, and the residue was taken up in dichloromethane/iso-propanol (3:1, 10 mL). The resulting suspension was filtered, and the filtrate was concentrated *in vacuo* to afford the corresponding hydrazide as a solid in 60–90% yield. **4–9** were synthesized according to GP1 and their spectral data correspond to those reported in the literature.[7–9]

General procedure for Boc-protected amino ester formation (GP2):

A solution of amino acid ester hydrochloride and Et_3N in anhydrous CH_2Cl_2 (45 mL) was added to a solution of di-*tert*-butyldicarbonate in CH_2Cl_2. The reaction mixture was stirred at r.t.for 2–3 h. The organic solution was washed with saturated aqueous NaCl solution, dried over Na_2SO_4 and filtered. The solvent was removed, and the crude was purified by flash chromatography (pentane/EtOAc) to afford the desired product in 52–81% yield as a white solid. **24** was synthesized according to GP2 and its spectral data correspond to those reported in the literature.[10]

General procedure for Boc-protected amino hydrazide formation (GP3):

Hydrazine monohydrate was added to a solution of Boc-protected amino ester in EtOH (10 mL). The reaction mixture was heated to reflux for 5 h. The reaction mixture was concentrated and water was poured onto the residue. After filtration, the solid was dried *in vacuo* to afford pure product in 70–98% yield.

General procedure for bis-acylhydrazone formation (GP4):

The hydrazide (1 eq.) was dissolved in anhydrous methanol, treated with isophthalaldehyde (**3**, 2 eq.), and the mixture was heated to reflux for 3 h. The reaction mixture was allowed to cool to r.t. and concentrated *in vacuo.* The resulting product was directly used in the following step. The residue was dissolved in anhydrous dichloromethane (10 mL), and HCl/diethyl ether (2 M, 10 eq.) was added. The mixture was stirred at room temperature for 3 h. The resulting precipitate was filtered and purified by reversed phase Prep HPLC (HPLC conditions: column, XTerra® Prep MS C18, 10 μ, 150 mm × 7.8 mm; flow rate, 1 mL min^{-1}; wavelength, 254 nm; temperature, 23 °C; gradient method, water/acetonitrile (0.1% TFA) 95:5 → 5:95 in 15 min (**13**) and 25 min (**16**).

(*R*/*S*)-Ethyl 2-(*tert*-butoxycarbonylamino)-3-(1H-indol-3-yl)propanoate (20)

The title compound was synthesized according to GP2 using **19** (3.0 g, 11.2 mmol), Et_3N (1.56 mL, 11.2 mmol) and di-*tert*-butyldicarbonate (2.3 g, 13.4 mmol). After purification, product **20** was obtained as a white solid (3.0 g, 81% yield). m.p. 153–158 °C; ^1H NMR (400 MHz; $CDCl_3$) δ=8.17 (s, 1H), 7.57 (d, *J*=8.0, 1H), 7.34 (d, *J*=8.0, 1H), 7.19 (t, *J*=8.0, 1H), 7.11 (t, *J*= 8.0, 1H), 6.99 (s, 1H), 5.09 (d, *J*=8.0, 1H), 4.63 (dt, *J*= 8.0, 5.4, 1H), 4.12 (q, *J*=6.9, 2H), 3.29 (t, *J*=4, 2H), 1.43 (s, 9H), 1.19 (t, *J*= 7.1, 3H); ^{13}C NMR (101 MHz, $CDCl_3$) δ=172.4, 155.4, 136.2, 122.8, 122.3, 119.7 (2C), 118.9, 111.2, 110.4, 79.9, 61.4, 54.4, 28.4 (3C), 28.1, 14.2; IR (cm^{-1}): 3319 (br), 2975, 1728, 1681, 1515, 1222, 1157, 735; HRMS (ESI) calcd for $C_{18}H_{25}N_2O_4^+$ $[M+H]^+$: 333.1809, found: 333.1806.

(*R*/*S*)-*Tert*-butyl 1-hydrazinyl-3-(1*H*-indol-3-yl)-1-oxopropan-2-ylcarbamate (21)

The title compound was synthesized according to GP3 using **20** (2.8 g, 8.42 mmol) and N_2H_4. H_2O (3.27 mL, 67 mmol). After purification, product **21** was obtained as a white solid (2.6 g, 98% yield). m.p. 200 – 203 °C; ^1H NMR (400 MHz, $(CD_3)_2SO$) δ=10.79 (1 H, s), 9.13 (s, 1H), 7.59 (d,

J=8.0, 1H), 7.31 (d, *J*=8.0, 1H), 7.12 (s, 1H), 7.05 (t, *J*=8.0, 1H), 6.97 (t, *J*=8.0, 1H), 6.76 (d, *J*=8.0, 1H), 4.30–3.95 (m, 3H), 3.07–2.77 (m, 2H), 1.31 (s, 9H); ^{13}C NMR (101 MHz, $(CD_3)_2SO$) δ=171.4, 155.1, 136.0, 127.3, 123.7, 120.8, 118.5, 118.2, 111.2, 110.2, 77.9, 53.7, 28.2(3C), 28.1; IR (cm^{-1}): 3335 (br), 2975, 2908, 1659, 1680, 1524, 1248, 1053, 736, 631; HRMS (ESI) calcd for $C_{16}H_{23}N_4O_3^+$ [*M*+H]$^+$: 319.1765, found: 319.1763.

(*S*)-*Tert*-butyl 1-hydrazinyl-1-oxo-3-phenylpropan-2-ylcarbamate (25)

The title compound was synthesized according to GP3 using **24** (1.80 g, 6.44 mmol) and N_2H_4. H_2O (2.5 mL, 51.6 mmol). After purification, the product **25** was obtained as a white solid (1.3 g, 70% yield). The spectral data correspond to those reported in the literature.[10]

***N*',*N*''*E*,*N*',*N*''*E*)-*N*',*N*''-(1,3-Phenylenebis(methan-1-yl-1-ylidene))bis(2-amino-3-(1H-indol-3-yl)propanehydrazide (13)**

The title compound was synthesized according to GP4 using **21** (405 mg, 1.27 mmol) and isophthalaldehyde (**3**, 85 mg, 0.64 mmol). After purification, product **13** was obtained as bis-trifluoroacetate salt as a white solid (50 mg, 1.2% yield). m.p. > 165 °C (decomposition); ^{1}H NMR (400 MHz, CD_3OD) δ=8.06 – 7.91 (m, 2H), 7.87 – 7.77 (m, 1H), 7.73 – 7.64 (m, 3H), 7.49 (dt, *J*=10, 8, 1H), 7.38 (d, *J*=8, 1H), 7.33 – 7.17 (m, 3H), 7.16 – 6.95 (m, 5H), 5.12 (dt, *J*=9, 6, 1H) ,5.03 (dt, *J*=9, 6, 1H), 4.31 – 3.97 (m, 1H), 3.60 – 3.03 (m, 4H). ^{13}C NMR (101 MHz, CD_3OD) δ=171.6, 167.5, 163.0, 162.5, 150.6, 147.0, 146.9, 146.8 (2C), 138.4, 138.3, 138.2, 135.8, 135.7, 135.7 (2C), 130.8, 130.3, 130.0, 129.7, 128.4, 128.3 (2C), 128.2, 127.7, 127.5, 127.2, 125.7, 125.5 (2C), 122.9 (2C), 120.3, 120.2 (2C), 119.1, 119.0, 112.7 (2C), 112.6, 108.3 (2C), 107.7, 54.4, 52.8, 52.7, 29.0, 28.4, 28.2. ^{19}F NMR (376 MHz, CD_3OD) δ=–76.9. IR (cm^{-1}): 3404 (br), 3224 (br), 3062 (br), 1670, 1199, 1134; HRMS (ESI) calcd for $C_{30}H_{31}N_8O_2^+$ [*M*+H]$^+$: 535.2564, found: 535.2556.

(2*S*,2'*S*,N',N''E,N',N''E)-N',N''-1,3-phenylenebis(methan-1-yl-1-ylidene))bis(2-amino-3-phenylpropanehydrazide (16)

The title compound was synthesized according to GP4 using **25** (1.0 g, 3.6 mmol) and isophthalaldehyde (**3**, 240 mg, 1.8 mmol). After purification, product **16** was obtained as bis-trifluoroacetate salt as a white solid (40 mg, 4% yield). m.p. 148 – 155 °C; ^{1}H NMR (400 MHz, (CD_3OD) δ=8.13 (s, 1H), 8.08 (s, 1H), 7.92 (s, 1H), 7.90 (s, 1H), 7.82 – 7.69 (m, 2H), 7.58 – 7.45

(m, 1H), 7.42 – 7.23 (m, 9H), 7.23 – 7.13 (m, 1H), 5.15 (t, J=8, 1H), 5.08 (t, J=8, 1H), 4.24 – 4.10 (m, 1H), 3.38 – 3.13 (m, 4H); ^{13}C NMR (101 MHz, CD_3OD) δ=171.2, 167.1, 163.0 (q, J=35), 151.5, 150.1, 147.4, 145.8, 136.0, 135.9, 135.8, 135.4, 131.4, 131.1, 130.9 (2C), 129.9, 129.5, 129.3, 128.3, 128.0, 119.8, 116.8, 113.9, 56.0, 54.5, 54.4, 53.0, 52.9, 40.1, 39.5, 38.8, 38.2. ^{19}F NMR (376 MHz, CD_3OD) δ=–76.9; IR (cm^{-1}): 3439 (br), 3039 (br), 2919 (br), 1669, 1199, 1131; HRMS (ESI) calcd for $C_{26}H_{29}N_6O_2^+$ [M+H]$^+$: 457.2346, found: 457.2341.

NMR spectra

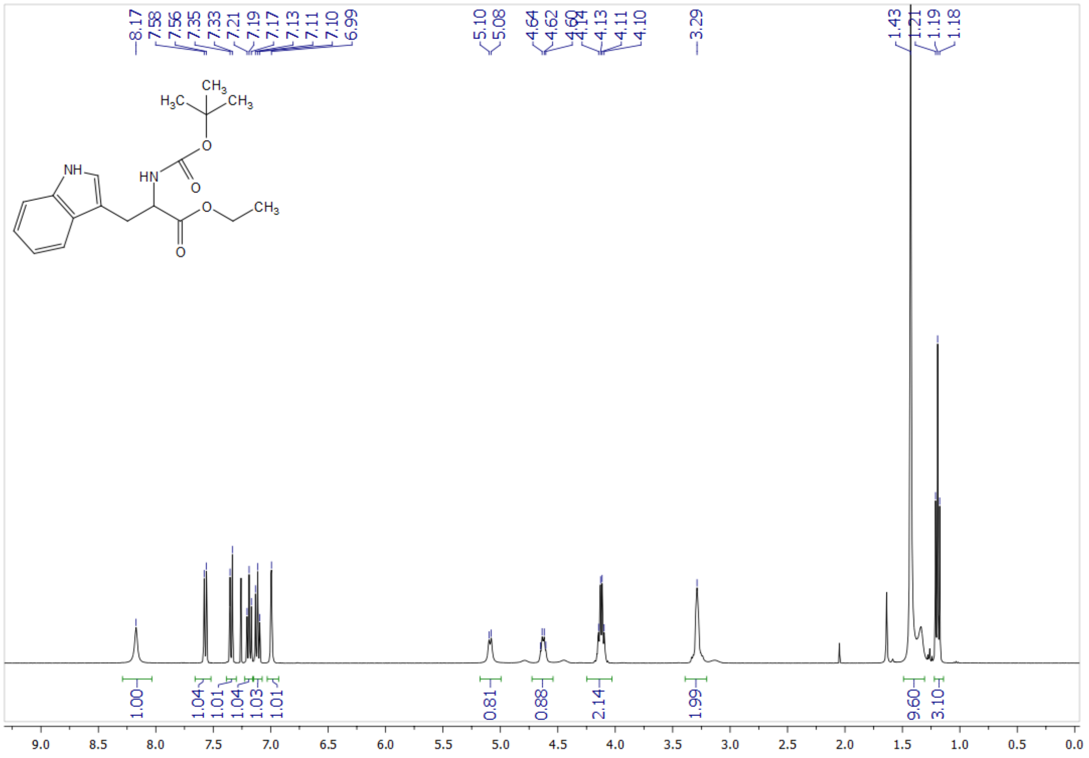

Figure S6. ^{1}H-NMR spectrum of **20**.

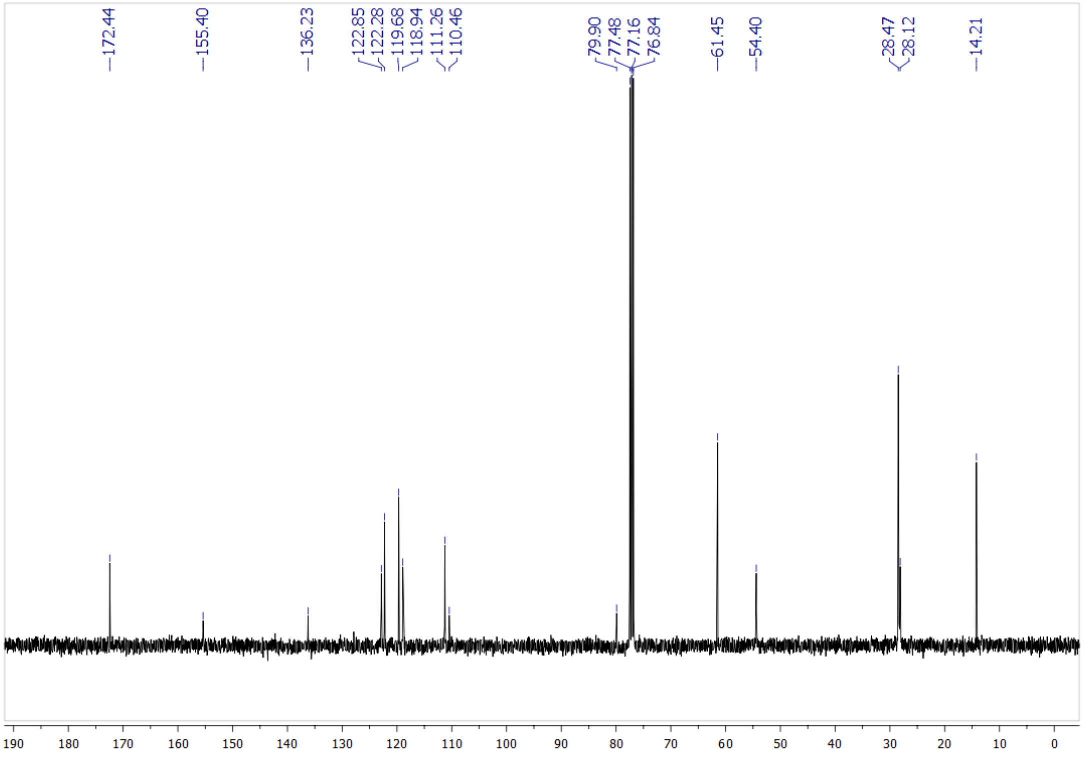

Figure S7. ^{13}C-NMR spectrum of **20**.

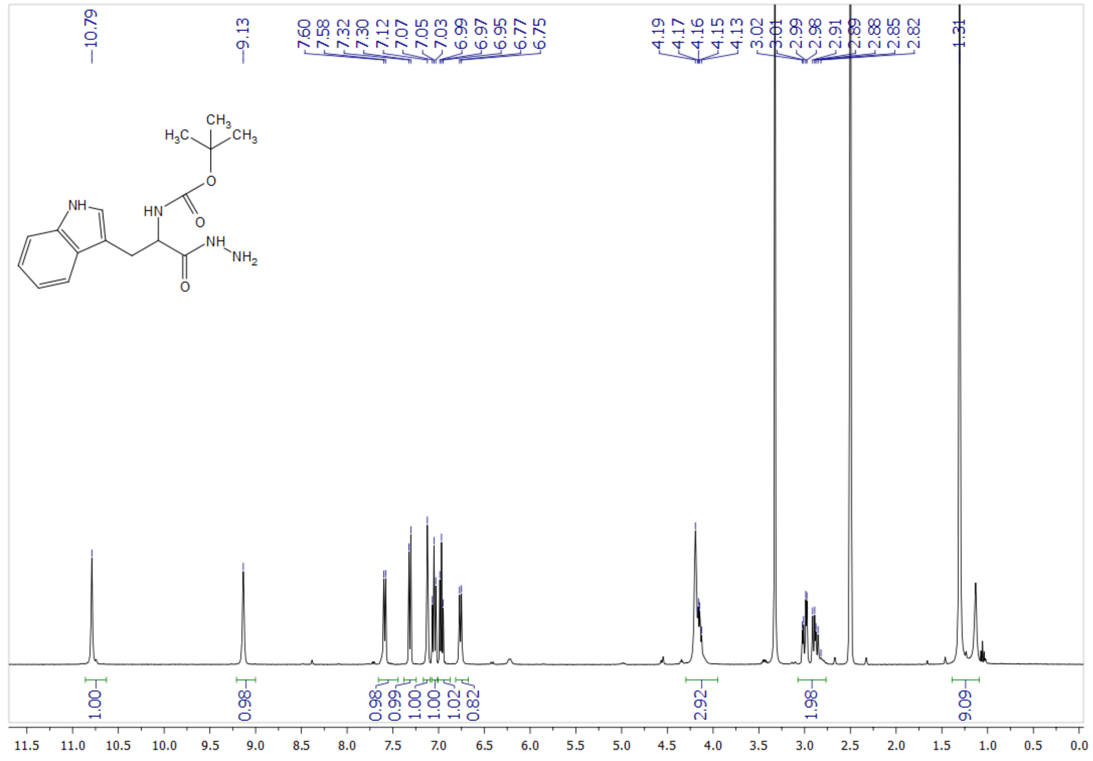

Figure S8. ^{1}H-NMR spectrum of **21**.

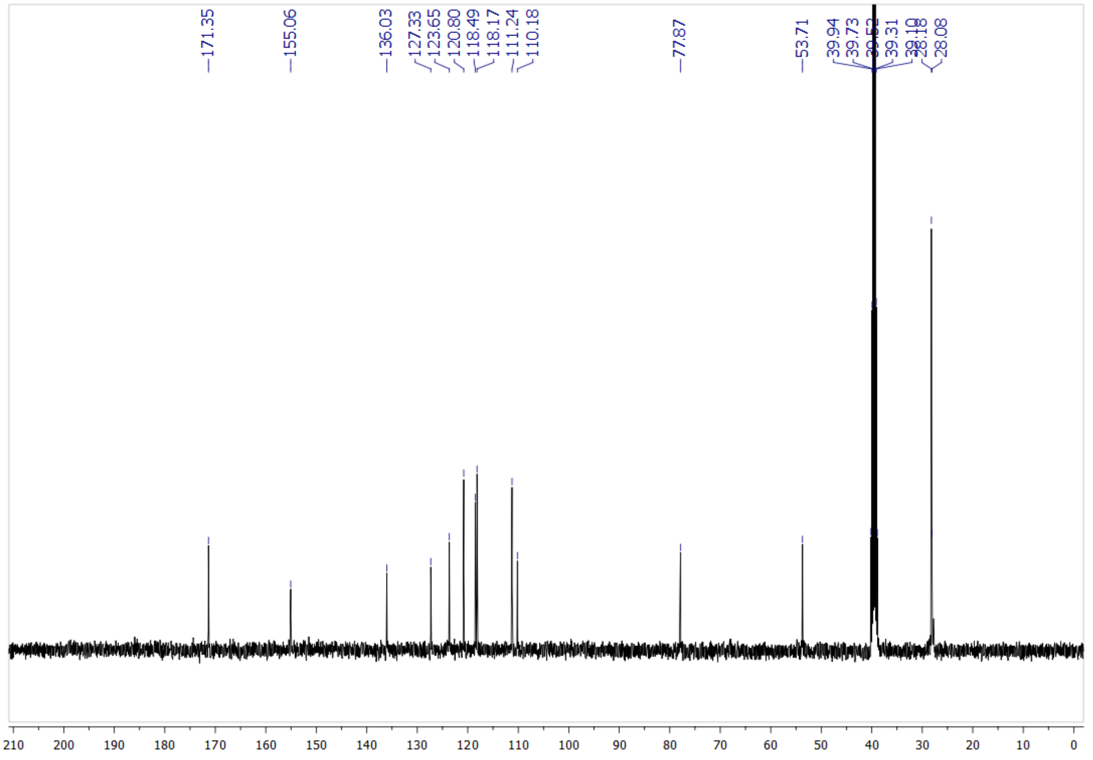

Figure S9. ^{13}C-NMR spectrum of **21.**

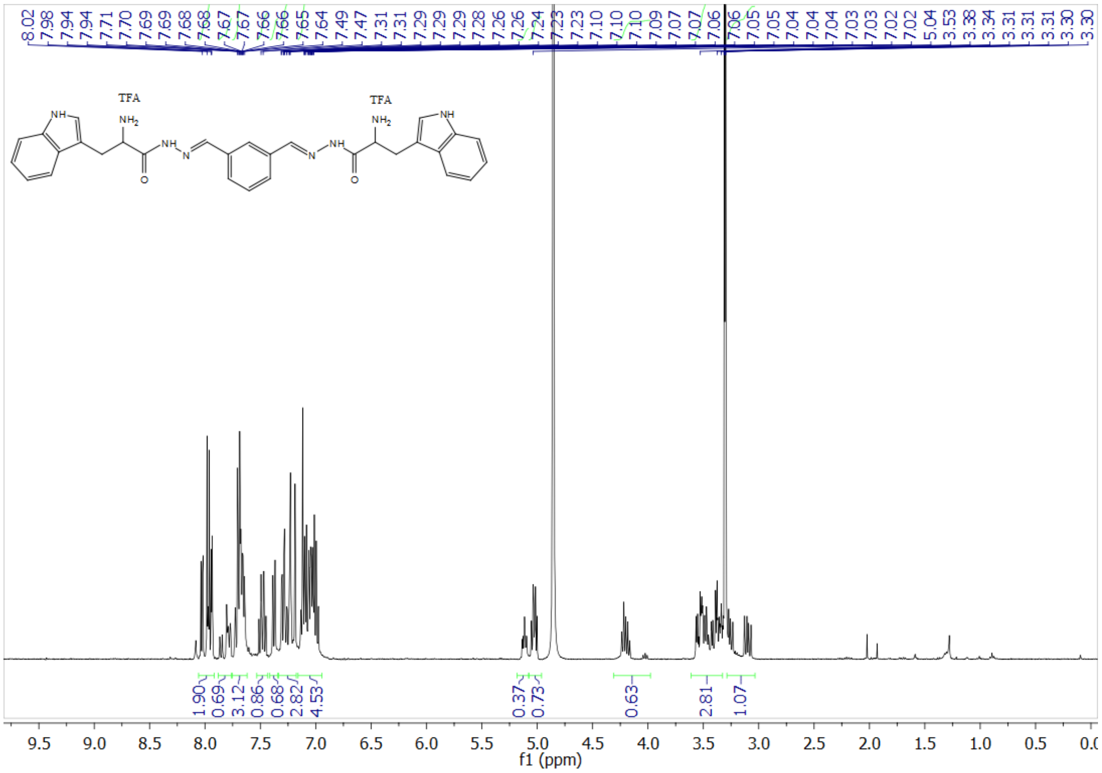

Figure S10. ^{1}H-NMR spectrum of **13**.

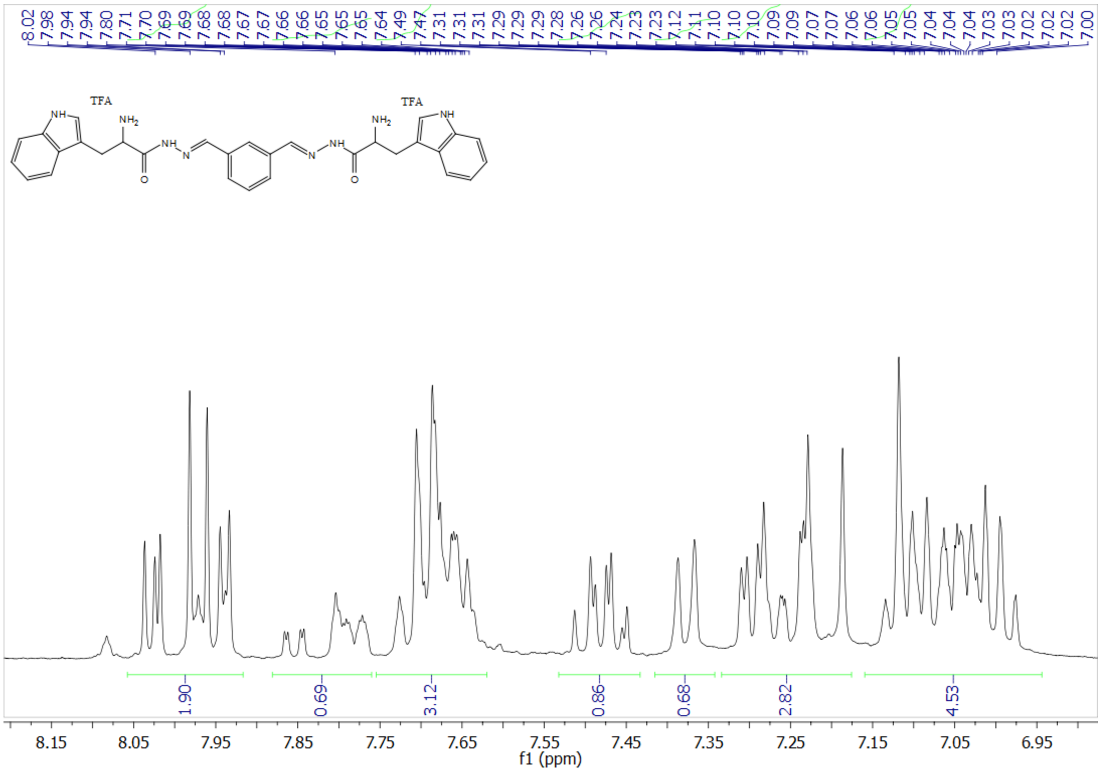

Figure S11. ^{1}H-NMR spectrum of **13** (expanded region).

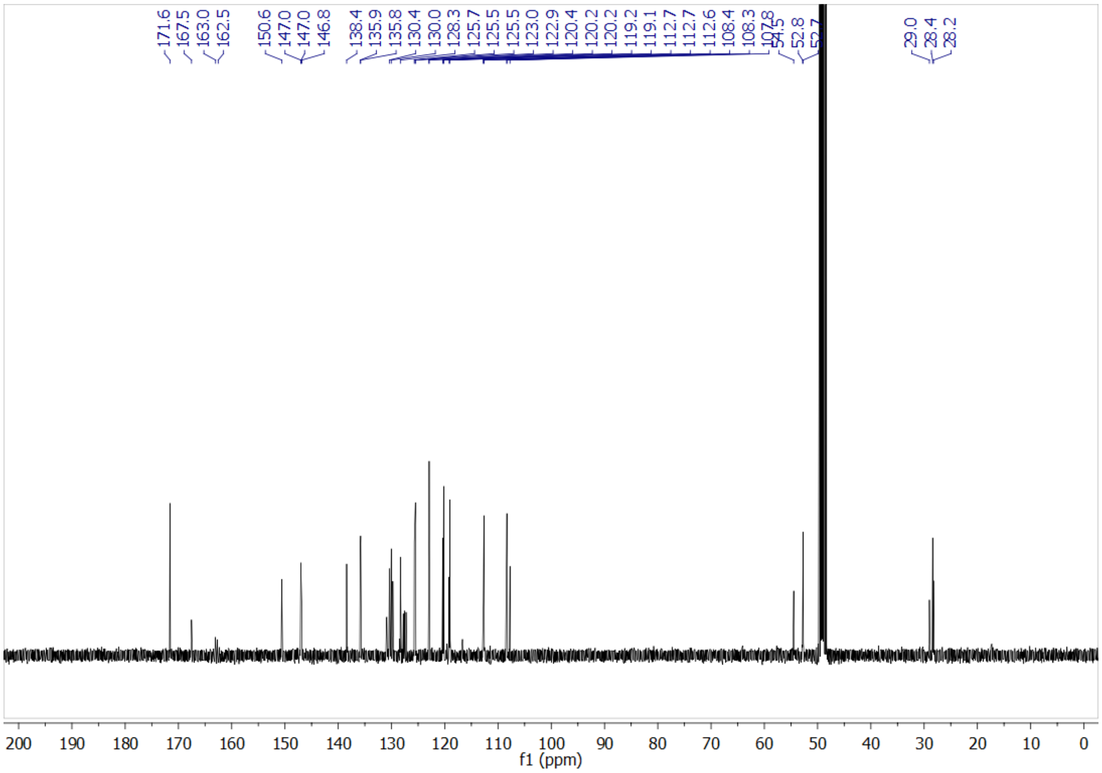

Figure S12. ^{13}C-NMR spectrum of **13**.

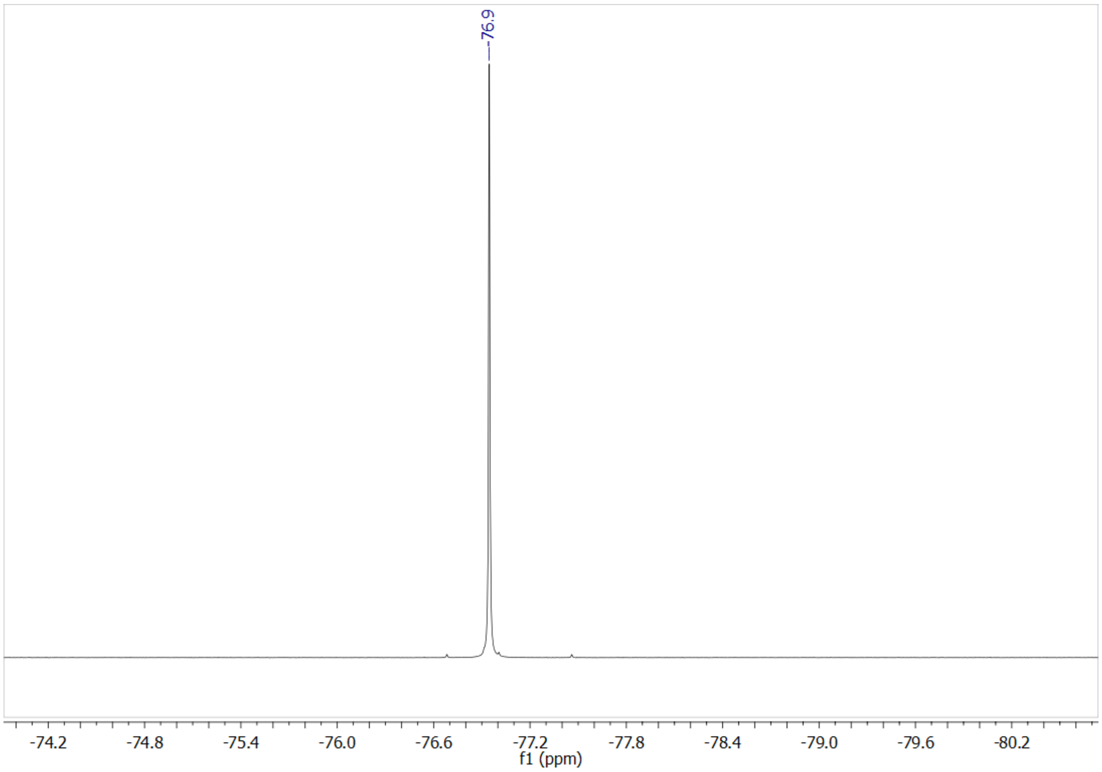

Figure S13. ^{19}F-NMR spectrum of **13**.

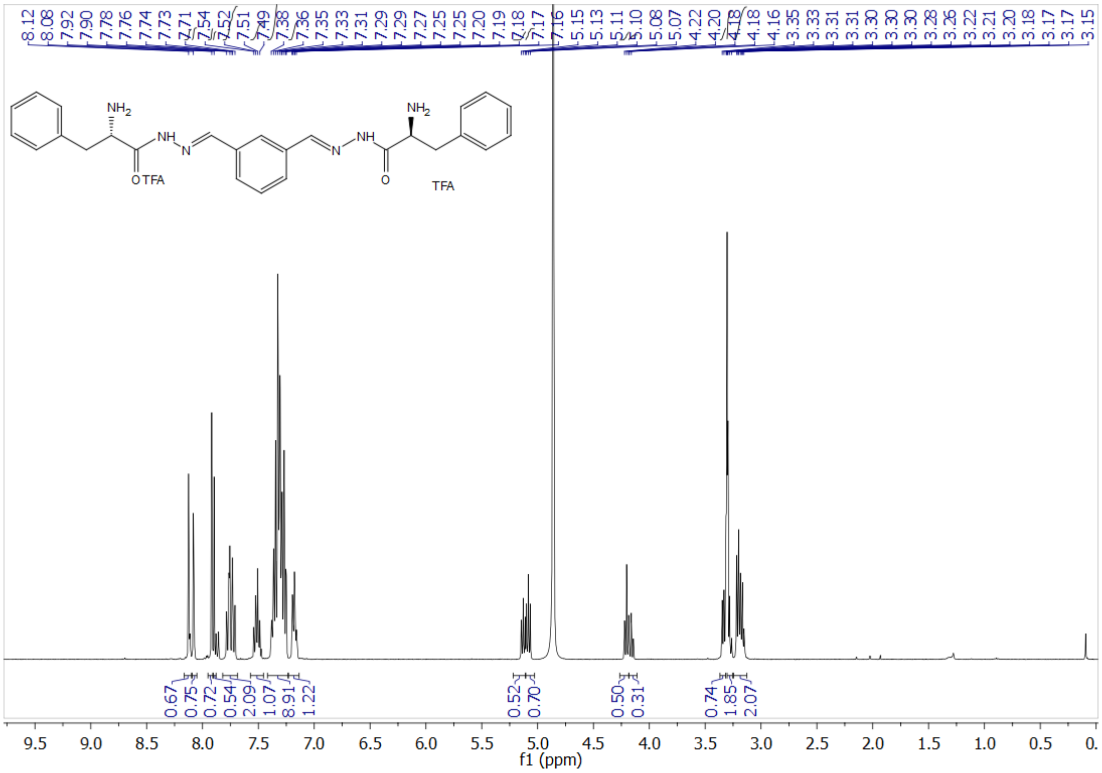

Figure S14. ^{1}H-NMR spectrum of **16**.

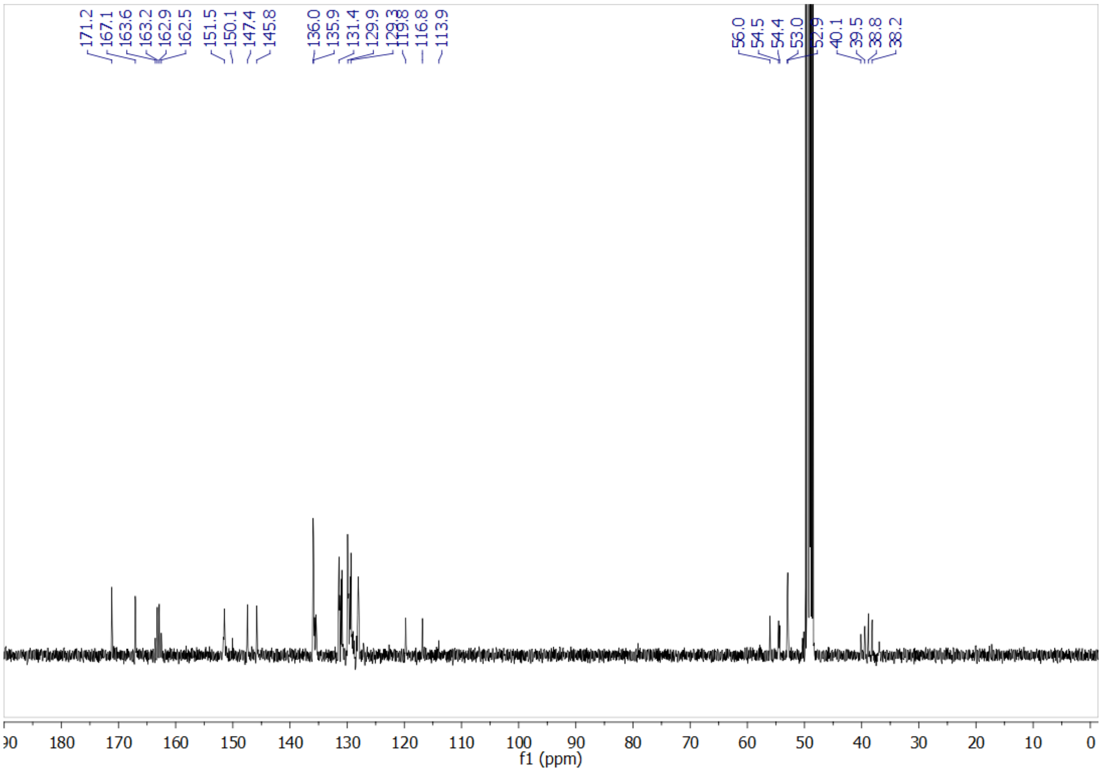

Figure S15. ^{13}C-NMR spectrum of **16**.

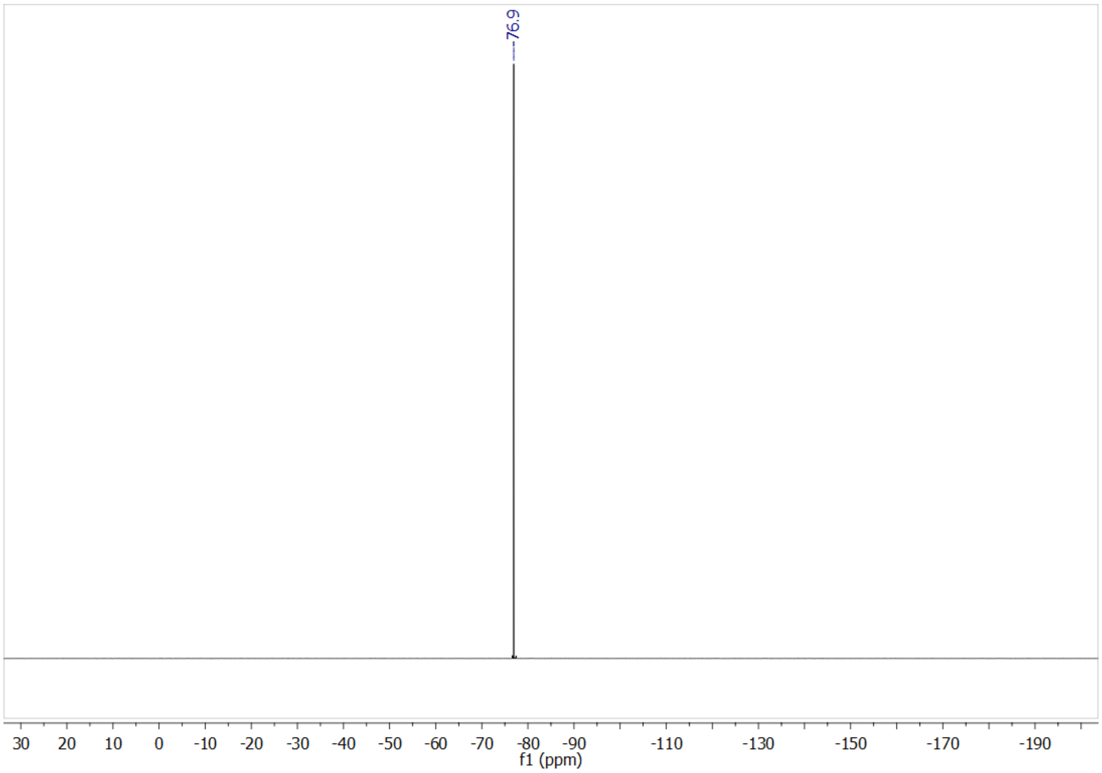

Figure S16. ^{19}F-NMR spectrum of **16**.

UPLC chromatograms

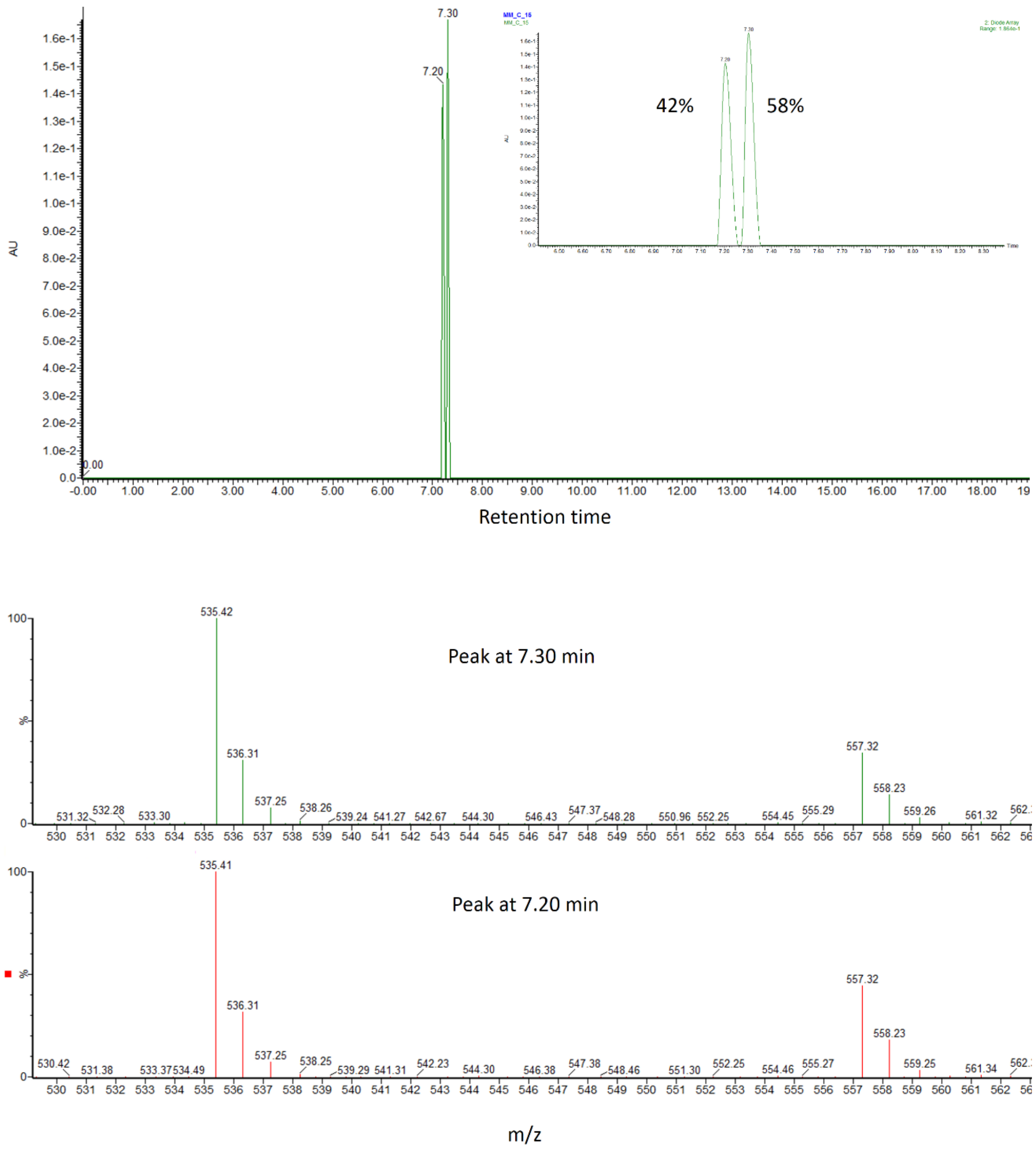

Figure S17. HPLC chromatogram of **13** and its mass analysis.

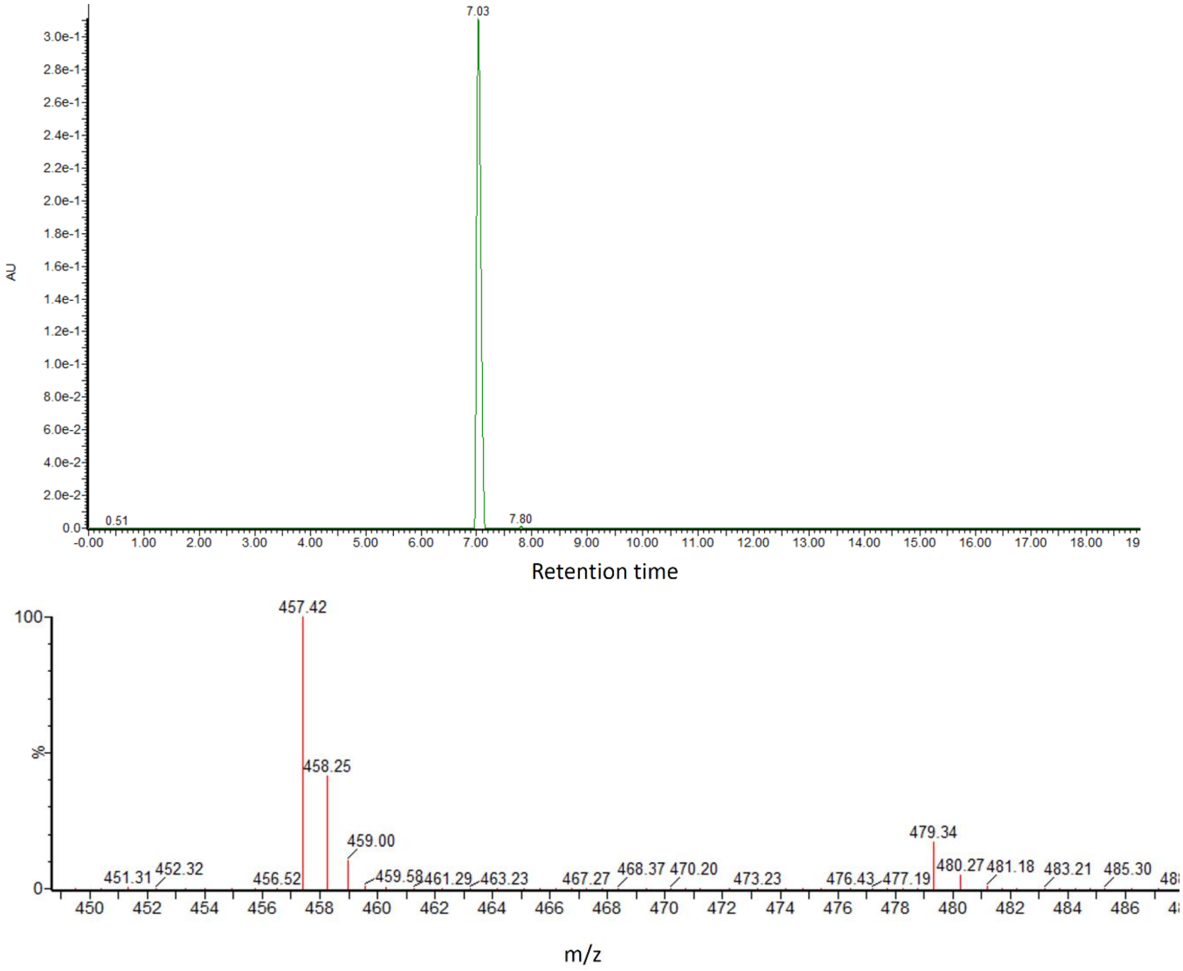

Figure S18. HPLC chromatogram of **16** and its mass analysis.

References

[1] M. Mondal, N. Radeva, H. Köster, A. Park, C. Potamitis, M. Zervou, G. Klebe, A. K. H. Hirsch, *Angew. Chem. Int. Ed.* **2014**, *53*, 3259–3263.

[2] PyMOL(TM) Molecular Graphics System, Warren L. DeLano, http://www.pymol.org/, version 1.4.1.

[3] M. K. Larson, J. R. Whitaker, *J. Dairy Sci.* **1970**, *53*, 253–261

[4] H. Köster, T. Craan, S. Brass, C. Herhaus, M. Zentgraf, L. Neumann, A. Heine, G. Klebe, *J. Med. Chem.* **2011**, *54*, 7784–7796.

[5] P. R. Gerber, K. Müller, *J. Comput. Aided. Mol. Des.* **1995**, *9*, 251–268.

[6] H. Gohlke, M. Hendlich, G. Klebe, *Perspect. Drug Discov. Des.* **2000**, *20*, 115–144.

[7] A. Kudelko, W. Zieliński, K. Ejsmont, *Tetrahedron* **2011**, *67*, 7838–7845.

[8] A. Kudelko, W. Zieliński, K. Jasiak, *Tetrahedron Lett.* **2013**, *54*, 4637–4640.

[9] V. Nalini, R. Girija, *Res. J. Chem. Environ.* **2013**, *17*, 12–17.

[10] C. F. Da Costa, A. C. Pinheiro, M. V. De Almeida, M. C. S. Lourenço, M. V. N. De Souza, *Chem. Biol. Drug Des.* **2012**, *79*, 216–222.
